# Joining the in vitro immunization of alpaca lymphocytes and phage display: rapid and cost effective pipeline for sdAb synthesis

**DOI:** 10.1186/s12934-017-0630-z

**Published:** 2017-01-23

**Authors:** Lubos Comor, Saskia Dolinska, Katarina Bhide, Lucia Pulzova, Irene Jiménez-Munguía, Elena Bencurova, Zuzana Flachbartova, Lenka Potocnakova, Evelina Kanova, Mangesh Bhide

**Affiliations:** 1 Laboratory of Biomedical Microbiology and Immunology, University of Veterinary Medicine and Pharmacy, 73, 04181 Kosice, Slovakia; 2 Institute of Neuroimunnology, Slovak Academy of Sciences, Bratislava, Slovakia

**Keywords:** VHH, In vitro immunization, OspA, Phage display, Single domain antibodies

## Abstract

**Background:**

Camelids possess unique functional heavy chain antibodies, which can be produced and modified in vitro as a single domain antibody (sdAb or nanobody) with full antigen binding ability. Production of sdAb in conventional manner requires active immunization of *Camelidae* animal, which is laborious, time consuming, costly and in many cases not feasible (e.g. in case of highly toxic or infectious antigens).

**Results:**

In this study, we describe an alternative pipeline that includes in vitro stimulation of naïve alpaca B-lymphocytes by antigen of interest (in this case endothelial cell binding domain of OspA of *Borrelia*) in the presence of recombinant alpaca interleukins 2 and 4, construction of sdAb phage library, selection of antigen specific sdAb expressed on phages (biopanning) and confirmation of binding ability of sdAb to the antigen. By joining the in vitro immunization and the phage display ten unique phage clones carrying sdAb were selected. Out of ten, seven sdAb showed strong antigen binding ability in phage ELISA. Furthermore, two soluble forms of sdAb were produced and their differential antigen binding affinity was measured with bio-layer interferometry.

**Conclusion:**

A proposed pipeline has potential to reduce the cost substantially required for maintenance of camelid herd for active immunization. Furthermore, in vitro immunization can be achieved within a week to enrich mRNA copies encoding antigen-specific sdAbs in B cell. This rapid and cost effective pipeline can help researchers to develop efficiently sdAb for diagnostic and therapeutic purposes.

## Background

Family *Camelidae* is in the spotlight in antibody engineering. Serum of camelids contain both conventional heterotetrameric antibodies and unique functional heavy (H)-chain antibodies (HcAbs), which were discovered in early 90 s [1]. This type of antibodies can form up to 75% of whole antibody repertoire [1]. Lack of C_H_1 in the H chain causes failure to pair with a light chain that results in the lower molecular weight (approx. 90 kDa) in comparison to conventional antibodies (approx. 150 kDa). VH regions of HcAbs, called VHH, are highly homologous with VH regions of conventional antibodies. However, mutational hotspots within VHH have been identified. Such hotspots are necessary for its stabilization, avoiding pairing with light chains and conferring high refolding ability [2]. The VHH regions can be amplified with PCR from HcAbs sequence to produce smaller antibody fragments (e.g. 15 kDa) with full binding ability. These small fragments are called nanobodies^®^ (Nbs) or single-domain antibodies (sdAb) [3].

sdAb consist only of VHH regions and are able to penetrate into difficult areas due to their small size or get through physical tissue that both HcAbs and conventional antibodies are not able to access [3]. sdAb can recognize unique epitopes, such as concave epitopes and thus have the possibility of succeeding in therapies where conventional antibodies commonly fail [4–8]. Moreover, sdAb have been successfully used also for diagnosis and inhibition of several types of cancer [9–12]. A huge advantage of using sdAb as therapeutics is the possibility of oral administration. By contrast, conventional antibodies have to be intravenously or subcutaneously injected. Harmsen et al. [13] successfully used sdAb orally to treat diarrhea in piglets. Beside medical applications, sdAbs are also used in research as tools for affinity chromatography [14], chromatin immunoprecipitations [15] or as crystallization chaperones in x-ray crystallography [16].

A conventional pipeline for nanobody synthesis includes active immunization of healthy *Camelidae* animals, extraction of mRNA from blood of immunized animals and ligation of VHH specific cDNA in phagemid followed by selection of antigen specific antibody by phage display [17]. Recently, a ribosome display was also employed for sdAb production as an alternative to phage display [18, 19]. A conventional pipeline has several disadvantages such as high cost for maintaining *Camelidae* animals and the comparatively longer period necessary for immunization. Furthermore, when production of sdAb towards multiple target antigens is desired, it would be necessary to maintain a large number of camelids.

Antigen-induced in vitro production of antibodies was suggested as an alternative method to generate conventional antibodies [20–22]. However, this alternative has never been used in the sdAb production pipeline. This method is based on the theory of spontaneous recombination of V-, D-, J- segments of antibodies in healthy B lymphocytes [23]. The co-cultivation of isolated B cells with target antigens triggers up-regulation of natural specific antibodies in an antigen-dependent manner [20]. It is important to note that, during the process of in vitro immunization interleukins (ILs, mainly IL-2 and IL-4) from the family *Camelidae* are essential for B cells activation and differentiation.

In the present study we describe a rapid pipeline for sdAb production that could replace the conventional technique which relies on the animal immunization. The antigen used in this study is an endothelial cell binding domain of OspA (outer surface protein A) of neuroinvasive *Borrelia*. This domain is also called as HUVEC (human umbilical vein endothelial cells) binding domain (thus hereafter designated as H-OspA). This protein is responsible for binding and translocation of *Borrelia* through the blood–brain barrier (BBB) [24, 25]. In the experimental pipeline, we immunized B cells in vitro with H-OspA, mRNA was isolated, reverse transcribed, gene fragment encoding VHH region was amplified and used to construct sdAb phage library. The library was screened to isolate antigen binding VHH fragment and antigen specific phage clones were sequenced. Based on sequence alignment, clones were grouped into ten families and representative of each family was tested for their binding affinity to antigen with dot-blotting, and phage ELISA. Furthermore, the clones with highest and lowest affinity were produced as soluble sdAb and their affinities were measured by bio-layer interferometry. The pipeline described here allows rapid and low-cost production of antigen specific sdAbs with minimal use of animals.

## Methods

### Synthesis of IL-2 and IL-4

**Fig. 1:**
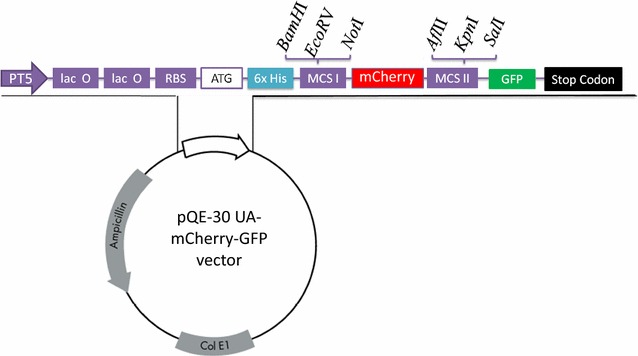
Vector map of pQE-30-mCherry-GFP plasmid (4880 bp). *PT5* T5 promoter, *lac O* lac operator, *RBS* ribosome binding site, ATG Start codon, *6xHis* His tag sequence, *MCS I/MCS II* multiple cloning sites; mCherry—red fluorescent protein that serve as stuffer, GFP, green flourescent protein; Stop codon; Col E1, Col E1 origin of replication; Ampicillin, ampicillin resistance gene. Note that incorporation of the stop codon at 3′ of insert produces recombinant protein without GFP fusion

Interleukins IL-2 and IL-4 of alpaca, necessary for in vitro immunization, were produced. In short, IL-2 and IL-4 coding sequences were retrieved from the GenBank (KM205215.1 and KM205216.1, http://www.ncbi.nlm.nih.gov/) and synthesized commercially (Invitrogen, Slovakia) with flanking sequences containing restriction sites for *BamH*I at 5′ and amber stop codon followed by *Sal*I at 3′ end. DNA fragments were digested with *BamH*I and *Sal*I (Thermo Fisher Scientific, Slovakia) and ligated into pQE-30-mCherry-GFP plasmid (Fig. 1, in-house modified vector pQE-30 UA, Qiagen). Please note that in this vector mCherry serves as stuffer sequence, which is cut out during the digestion of vector with restriction enzymes, whereas incorporation of amber stop codon at 3′ of IL gene ensures no fusion of GFP to ILs. Ligation mix was purified using NucleoSpin (Macherey–Nagel, Germany) and transformed into *E. coli* SG13009 for IL-2, and M15 strain for IL-4 (Qiagen, Germany). Transformants were selected from LB agar plates (lysogeny broth agar, 10 g/L tryptose, 10 g/L NaCl, 10 g/L yeast extract, 2% bacteriological agar) supplemented with 1% glucose (G), 5 µg/mL Kanamycin (K) and 5 µg/mL Carbenicillin (C). Presence of IL-2 or IL-4 encoding gene in transformants was confirmed by sequencing (vector specific primers UA Insertom F and R, presented in Table 1).

**Table 1:** Sequences of primers used in the PCR

Primer	Sequence 5′–3′	Method
sDAb-Not-R	CCAGCGGCCGCTSWGGAGACRGTGACCWGGGTCC	Reverse transcription of RNA; VHH amplification
sDAb-NcoAsc-F	CGGCCATGGCCGGGCGCGCCGCCSAGGTGSAGSTSSWGSMGTC	VHH amplification
pSex Insertom F	ATGAAATACCTATTGCCTACGGCAG	Control PCR for electroporation
pSex Insertom R	CTACAACGCCTGTAGCATTCCAC	Control PCR for electroporation
UA Insertom F1	CGCATCACCATCACCATCACG	Control PCR for electroporation, plasmid pQE-30-mCherry-UA-GFP
UA Insertom R1	ACCAAATTGGGACAACACCAGTG	Control PCR for electroporation, plasmid pQE-30-mCherry-UA-GFP
sDAb-BamHI-F	AATGGATCCSAGGTGSAGSTSSWGSMGTC	VHH amplification for production of soluble sdAb
sDAb-SalI-R	GCTGTCGACCTATSWGGAAGACRGTGACCWGGGTCC	VHH amplification for production of soluble sdAb
H-OspA F	AGGATATCTAACGAAAAGGGTGAAACA	For production of H-OspA
H-OspA R without Stop	ATAGTCGACTTCTATGTCAGAGTCATCAAGTGC	For production of H-OspA with GFP fusion
H-OspA R with Stop	ATAGTCGACTCATTCTATGTCAGAGTCATCAAGTGC	For production of H-OspA without GFP fusion

### Purification of interleukins

Clones were cultivated in Terrific broth (15 g/L tryptose, 30 g/L yeast extract, 12.5 g/L NaCl, 2.5 g/L MgCl2/MgSO4, 100 µl/L metal mix, 7.5 mL/L glycerol) supplemented with 1% glucose, 5 µg/mL Carbenicillin and 5 µg/mL Kanamycin (TB/G/K/C) until OD_600_ = 6. Bacterial cells were pelleted (centrifugation at 6000 × *g* for 10 min) and resuspended in fresh TB medium without glucose (TB/K/C). Protein expression was induced with 1 mM IPTG (Fermentas, Slovakia) for 8 h at 20 °C. After induction, cells were pelleted (17,880 × *g* for 10 min) and lysed in lysis buffer (0.03 M Na_2_HPO_4_, 0.5 M NaCl, 0.001% Tween 20, 10% glycerol) with four freeze–thaw cycles followed by sonication (2 cycles; 30-s pulses, 100% amplitude). His tagged- ILs were purified with nickel affinity chromatography (Ni–NTA agarose beads, ABT, Spain) as per manufacturer´s instructions. Eluted fraction was evaluated by SDS-PAGE and Western blotting using anti-His probe 1:3500 (Thermo Fisher Scientific). Molecular mass of purified proteins was identified on MALDI–TOF MS as described by Mlynarcik et al. [24]. Aliquots of purified ILs were stored at −20 °C either in PBS or in PBS containing 20% glycerol until use.

### Assessment of toxicity of interleukins and cell proliferation

Peripheral blood mononuclear cells (PBMCs) were cultured as described previously [25, 26]. Cells were incubated for 24 h (37 °C, 95% humidity, and 5% CO_2_) in the presence of 1 ng/µL of each IL, with or without 20% glycerol. This concentration of ILs was used previously for in vitro immunization of human B cells [26]. Cell cultures without ILs were run alongside as negative controls. Cell viability/proliferation was assessed with XTT proliferation test according to manufacturer’s instructions (Panreac Applichem, Germany). In brief, 100 µl of each cell suspension was transferred to 96-well plate and 50 µl of XTT solution was added. Cells were incubated for 2 h and OD was measured by Fisher Scientific™ accuSkan™ FC Filter-Based Microplate Photometer (Thermo Fisher Scientific) at 475 nm with reference absorbance at wavelength 660 nm. The proliferation (d) was measured as follows d = (a − b) − c; where in a—absorbance at 475 nm, b—absorbance at 660 nm and c—absorbance of blank medium (reference absorbance at 660 nm was also subtracted for c). Measurements were performed in quadruplicate. The assay was repeated three times.

### Production of H-OspA

Briefly, sequence encoding H-OspA was amplified by PCR from genomic DNA of SKT–7.1 strain of *Borrelia bavariensis* using primers depicted in Table 1. Purified PCR products were digested with restriction enzymes *EcoR*V and *Sal*I (Thermo Scientific), ligated into previously digested pQE-30-mCherry-GFP plasmid. To produce his-tagged H-OspA without GFP fusion we incorporated stop codon in antisense primer (Table 1, primer: H-OspA R with Stop), whereas, for the production of his-tagged OspA with C-terminal GFP fusion antisense primer was without stop codon (Table 1, primer: H-OspA R without Stop). Transformants were selected from LB agar plates supplemented with 1% glucose (G), 5 µg/mL Kanamycin (K) and 5 µg/mL Carbenicillin (C). Proteins were overexpressed with 1 mM IPTG for 16 h at 20 °C. Purification of proteins in native state was performed as described above.

### In vitro immunization of B cells

100 mL of heparinized blood was collected from 4 years old healthy alpaca. PBMCs were immediately isolated by density centrifugation using Histopaque medium (Sigma-Aldrich, Germany) according to manufacturer´s instructions. PBMCs in the buffy coat were transferred into a new tube and washed with eRDF medium (RPMI:DMEM:F12 in the ratio of 2:1:1 as described in [27]). PBMCs were pelleted (400×*g*, 20 min) and resuspended in 5 mL of 20 mM Leu–Leu methyl-ester hydrobromide prepared in eRDF (LLME) (Sigma-Aldrich). Cells were incubated for 20 min at room temperature and harvested by centrifugation (400×*g*, 20 min). Cell pellet was washed with eRDF medium and again resuspended in 1 mL of eRDF. Cell density was measured by BD Accuri C6 flow cytometer (BD Biosciences, USA) and adjusted to 1 × 10^6^ cells/mL using eRDF. Cell suspensions (2 mL/well) were incubated overnight in 12-well plates (37 °C, 95% humidity, 5% CO_2_) with 1 ng/mL of each IL, 0.25 µM Class A CpG oligonucleotide ODN 2216 (InVivoGen, USA) and 20 µL/mL Mycokill (PAA Laboratories, Germany). After incubation, antibody production was induced by adding 10 µg/mL of his tagged H-OspA (antigen). Cells were incubated for 24 h before adding 0.25 µM Class B CpG oligonucleotide ODN 2006 (InVivoGen, strong activator of B lymphocytes with weak stimulation of IFN-α secretion) to each well, and the incubation was continued until 72 h. Cell viability was checked every day under the microscope and cells count (proliferation) was performed by flow cytometry (BD Accuri C6 flow cytometer) using 2 µl of cell culture resuspended in 20 ml of fresh eRDF. After 72 h incubation, total RNA was isolated from the cells using PureZol (Bio-Rad, USA) and treated with DNaseI (Thermo Fisher Scientific) according to manufacturer’s instructions.

### VHH amplification

**Fig. 2:**
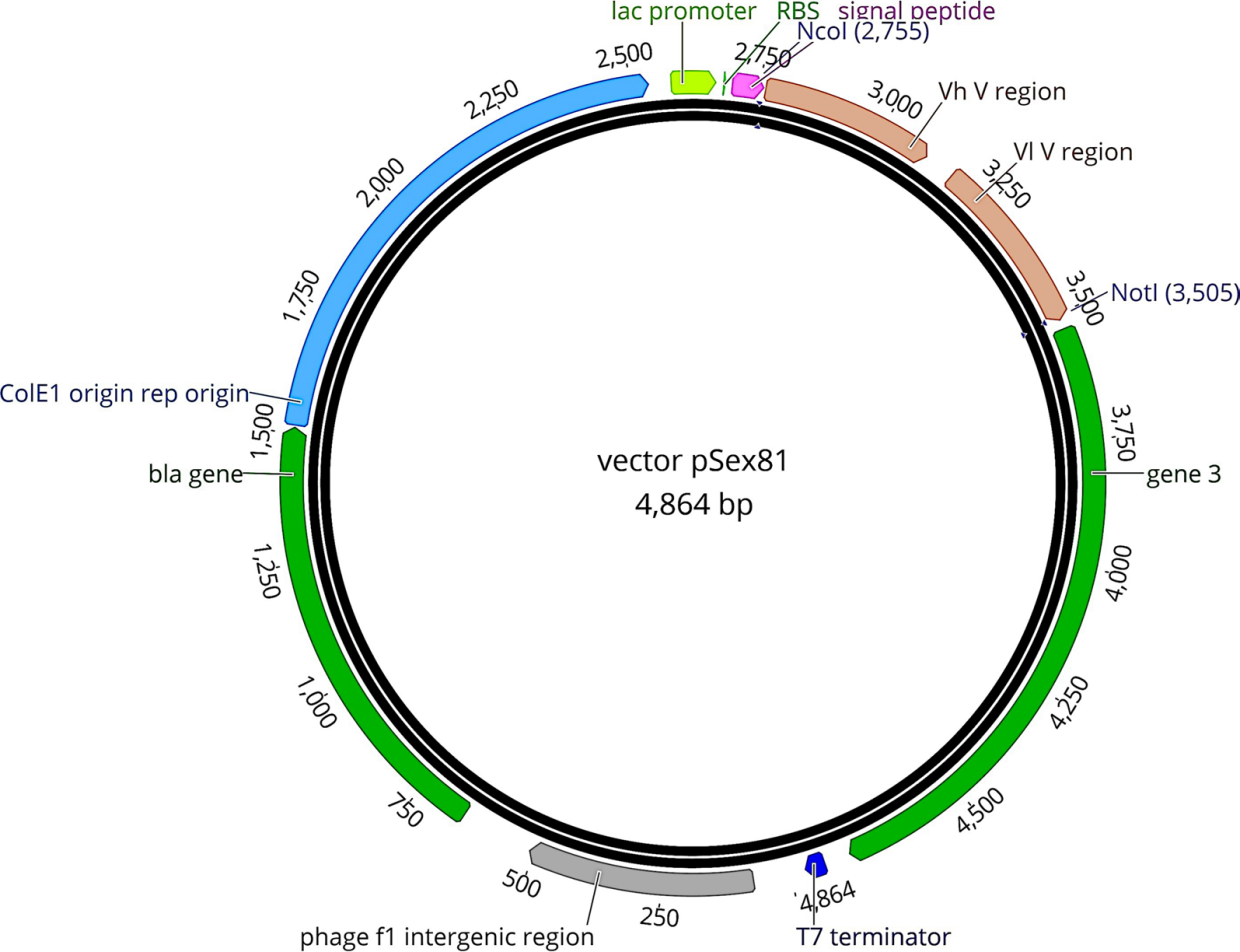
Vector map of pSex81 plasmid (4864 bp). RBS, ribosome binding site; Signal peptide—pel-B leader sequence; Vh V and VI V regions, scFv single-chain fragment variable that serves as stuffer which is replaced by VHH sequence encoding sdAb; gene 3, gene for pIII surface protein. Note that sdAb are fused to pIII. T7 terminator; Bla gene, β-lactamase gene which gives ampicillin resistance; Col E1, Col E1 origin of replication

cDNA was reverse transcribed from RNA using RevertAid reverse transcriptase (Thermo Fisher Scientific) and sDAb-Not-R primer (Table 1) according to manufacturer’s instructions. VHH were PCR amplified using gene-specific primers that amplifies sequence between Framework 1–4 (sDAb-NcoAsc-F and sDAb-Not-R; Table 1). Amplicons were purified by NucleoSpin (Macherey–Nagel, Germany) and digested with restriction enzymes *Nco*I and *Not*I (Thermo Fisher Scientific) for 1 h at 37 °C as per manufacturer´s instructions. Digested DNA fragments were column purified and ligated into the phagemid pSex81 (Fig. 2, Progen, Germany) and transformed into *E. coli* XL-1 blue (New England Biolabs, Germany). This ligation allows fusion of VHH with pIII protein of the phage. Transformants were selected randomly from LB agar plates (containing 5 µg/mL of Carbenicillin) and subjected for the sequencing using vector specific primers pSex Insertom F and R (Table 1) on ABI 3100 Avant sequencer (3.1 big-dye terminator kit, thermo scientific) to analyze the diversity of the library. The diversity was determined by distance matrix created in Geneious pro R9 (Biomatters LtD. New Zealand). All *E. coli* colonies were scraped in 10 mL of 50% glycerol in LB medium and stored at −20 °C for further experiments (this suspension of *E. coli* is referred to as a library, which contains large repertoire of VHH variants cloned into pSex81 backbone).

### Phage display

Phage display was conducted as described before [28]. In brief, 100 µL of the library was grown in 2 × TY medium (16 g/L tryptose, 10 g/L yeast extract, 5 g/L NaCl, pH 7) supplemented with 5 µg/mL Tetracycline and 1% glucose up to OD_600_ = 0.5. Cells were superinfected with helper phage M13K07 (20 phages/cell, Progen Biotechnik, Germany) for 1 h at 37 °C (30 min without shaking followed by 30 min with shaking at 250 rpm). Superinfected cells were incubated overnight in 2 × TY medium supplemented with 5 µg/mL Tetracycline, 5 µg/mL Carbenicillin and 5 µg/mL Kanamycin to allow phage escaping. Phages were precipitated with polyethylenglycol (20% PEG, 2.5 M NaCl). Number of phages were calculated by titration as described by Thie [28] on agar plates containing 2 × TY medium supplemented with 1% glucose, 5 µg/mL Tetracycline, and 5 µg/mL Carbenicillin. The selection (biopanning) of phages expressing antigen specific sdAb on pIII protein was performed by affinity chromatography. In short, precipitated phages were resuspended in phage dilution buffer (10 mM TrisHCl, 20 mM NaCl, 2 mM EDTA) and 2 × 10^11^ of phage particles were incubated with metal affinity (Co++) magnetic beads (Bruker, Germany) for 1 h at room temperature. Beads were spun and supernatant was recovered. This step removes the phages that bind nonspecifically to magnetic beads. The supernatant containing phages was then incubated with antigen immobilized on the metal affinity (Co++) magnetic beads at 4 °C overnight with constant shaking. Magnetic beads were washed 10 times as follows: first washing with PBS supplemented with 0.1% Tween20 (PBS-T) for overnight at 4 °C followed by eight washings with PBS-T (each for 2 min at 4 °C), and the last washing with PBS for 2 min at 4 °C). Before each washing beads were transferred to new tube to avoid carryover of the phages that possess affinity to the plastic. Antigen specific sdAb were eluted by PBS containing 10 µg/mL trypsin (pH 7.4, Promega, USA). Number of eluted phage particles were calculated by titration as described above. Three rounds of biopanning were performed and clones from last titration plate were used for amplification of phage clones.

### Amplification of phage clones

Twenty clones were picked randomly from the LB plates used in the last titration, sequenced with vector specific primers (pSex Insertom F and R, Table 1) as described above. VHH sequences were aligned (Geneious pro 9.0) and grouped based on sequence similarity. Representative *E. coli* clone from each group were amplified and phages were escaped by superinfection and precipitated as described before. Protein concentration of the phage pellet was measured by Bradford method and the concentration was set to approximately 50 ng/µl with phage dilution buffer. Phages were stored at −80 °C until their use in phage dot blotting.

### Qualitative phage dot blotting

Phage dot blot was performed to assess affinity of sdAb clones to H-OspA (antigen). In short, PVDF membrane (Millipore, USA) was first pre-wetted in methanol and then in PBS, and 2 µL of each diluted phage clone expressing sdAb was spotted in duplicate on two separate membranes (one membrane was used as input control to confirm presence of phages on membrane—phage input control, and second membrane was used for phage dot blot). Membranes were blocked in Odyssey blocking buffer (LI-Cor, USA) for 1 h at room temperature. Membrane for phage input control was incubated with anti-pVIII antibody (1:1000 GE Healthcare, United Kingdom), washed two times with PBS-T and the interaction was detected by IRDye detection reagent (LI-Cor, USA). The second membrane with phages (kept for phage dot blot) was incubated with GFP tagged H-OspA for 1 h at room temperature. After three washings with PBS-T, the interaction was detected by anti-GFP antibody conjugated with C770 IRdye (1:20,000 Biotium, USA). Signals were captured on LI-Cor Odyssey CLx (LI-Cor, USA). The experiment was repeated three times.

Another control was kept in the experiment to show that anti-pVIII antibody used in this experiment detects phage coat. For this helper phage MK13K07 (which has no VHH sequence) was spotted on the membrane, blocked in Odyssey blocking buffer and then incubated with anti-pVIII antibody. After washings with PBS-T interaction was detected by IRDye detection reagent.

To confirm that neither GFP tagged H-OspA, nor anti GFP antibody bind non-specifically to helper phage coat proteins, MK13K07 was spotted on the membrane and the membrane was included in phage dot blotting described above.

For an antigen input control, GFP tagged H-OspA binding domain was spotted on the separate membrane and presence of tagged protein was detected by anti-GFP antibody conjugated with C770 IR dye (1:20,000, 1 h incubation at room temperature, Biotium, USA).

### Quantitative phage ELISA

Phage ELISA was performed to measure affinity of selected sdAb clones to H-OspA. In brief, 96-well plate (Nunc, Denmark) was coated with 100 μL his-tagged H-OspA (no GFP tag) and a non-related protein (his-tagged serum amyloid A of *Salmo salar* fused with GFP; SAA; previously produced in our lab [2]) in carbonate/bicarbonate buffer (15 mM Na_2_CO_3_, 35 mM NaHCO_3_, pH = 9.6) at concentration of 50 μg/mL at 4 °C overnight. Wells were blocked by PBS-T for 1 h with gentle agitation. The plate was subsequently washed three times with PBS-T and 100 μL of phage clones (approx. 100 ng, diluted in PBS-T) were added to each well and incubated for 1 h at room temperature with gentle agitation. After three washings with PBS-T, 100 μL of anti-pVIII antibody (1:1000) was added to the wells, and the plate was incubated for 1 h at room temperature. After three washings, secondary anti-Mouse antibody conjugated with HRP (GE Healthcare, 1:1000) was added and the plate was incubated for 1 h at room temperature. The plate was washed three times and 100 μL of ELISA-HRP substrate 680 (Li-Cor) was added, and the plate was incubated in dark for 15 min. Twenty-five microliters of stop solution was added to each well and the plate was incubated for another 5 min in dark. Finally, the plate was read at 700 nm on Li-Cor Odyssey CLx. The assay was performed in triplicate.

To assess unspecific binding of phage clones to plastic, H-OspA and SAA proteins were excluded from above experiment. To rule out any non-specific binding of anti-pVIII and anti-Mouse antibodies to H-OspA, three wells coated with H-OspA were directly incubated with primary and secondary antibodies.

### Production of soluble sdAbs

DNA was extracted from *E. coli* clones infected with PhC11 and PhC12. The sequences encoding VHH were amplified by primers with *BamH*I overhang in sense and *Sal*I in antisense (Table 1). Please note that antisense primer contained stop codon downstream to *Sal*I restriction site. Amplified DNA was digested, ligated into pQE-30-mCherry-GFP, and transformed into *E. coli* SG130009. The soluble sdAb were overexpressed in TB medium containing 1 mM IPTG (16 h at 20 °C), and purified under native conditions using nickel affinity chromatography (Ni–NTA agarose beads, ABT, Spain) as per manufacturer´s instructions. The presence of sdAbs and their purity was checked by SDS-PAGE.

### Bio-layer interferometry

Bio-layer interferometry was performed on BLItz system (fortéBIO, USA) as per manufacturer’s guidelines. Streptavidin-coated Dip-and-Read Biosensors (fortéBIO) were equilibrated by assay buffer (PBS-T, 0.02% Tween-20) for 60 s. H-OspA, freshly biotinylated by EZ-Link™ Sulfo-NHS-SS-Biotin (ThermoFisher Scientific) according to manufacturer’s instructions, was subsequently bound to the sensor for 150 s at concentration of 250 μg/mL. Free surface of biosensor was blocked by biocytin (Sigma-Aldrich, 10 μg/mL) for 90 s. The sensor was then washed with assay buffer for 60 s. The affinity of soluble sdAb was measured in three different concentrations (7.25, 14.5, and 29 μM) for 240 s (120 s for association step and 120 s for dissociation step). For blank measurement, the biosensor was coated with H-OspA and association–dissociation steps were performed with assay buffer without sdAb. To evaluate the affinity constant K_D_ (equilibration between constants of association rate—K_a_ and dissociation rate—K_d_) of the sdAb, blank measurement was subtracted from the measurements of the sdAb, and global fitting (1:1) was used. Blitz Pro 1.1.0.31 software (fortéBio) was used to measure K_D_s.

## Results

### Production of IL-2 and IL-4

**Fig. 3:**
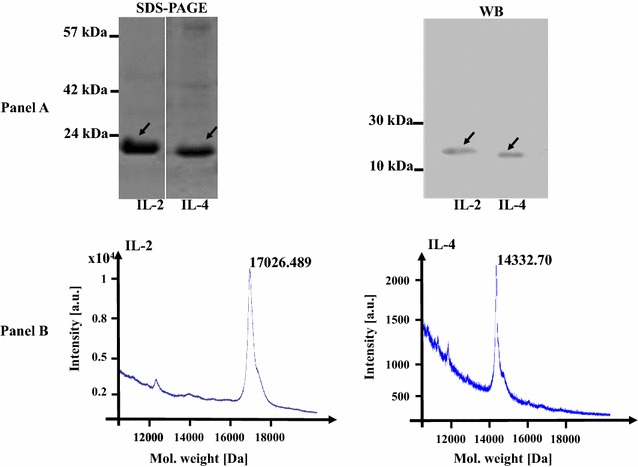
Purity of ILs was assessed by SDS-PAGE and Western blotting using anti-His probe (**a**). *Arrows* in **a** indicates ILs. Molecular mass of the ILs was also confirmed by MALDI–TOF (**b**). Masses 17.026 kDa of IL-2 and 14.332 kDa of IL-4 exactly matched with their predicted molecular weights (in silico prediction in Geneious Pro software)

Interleukins 2 and 4 of *Vicugna pacos* (alpaca) are not commercially available. Although ILs from other species (like *sus scrofa*) are available, their ability to proliferate/activate cross species B cells is doubtful. Thus we decided to produce recombinant ILs of alpaca. Synthetic genes encoding IL-2 and IL-4 were ligated into the expression vector, transferred into *E. coli* strains and proteins were overexpressed with IPTG. Interleukins were purified in native state with nickel affinity chromatography. Approximately 61 mg of IL-2 per liter of LB medium and 35 mg of IL-4 per liter were obtained. The purity of ILs was verified by SDS-PAGE and Western blot using anti-His probe (Fig. 3, panel A). The molecular weights of his-tagged ILs were confirmed on the MALDI–TOF, which were in accordance with the masses predicted in silico (17 kDA for IL-2 and 14 kDa for IL-4; Fig. 3, panel B).

### Recombinant ILs showed cell proliferation and non-toxicity

**Fig. 4:**
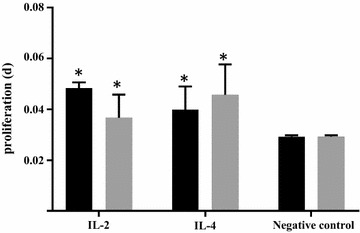
PBMCs were cultured with (IL-2 and IL-4) or without interleukins (negative control) to assess any possible cytotoxicity of recombinant ILs as well as to confirm their ability to induce lymphocyte proliferation (d). Proliferation assay was performed using XTT. ILs were used with (*gray columns*) or without glycerol (*black columns*) to rule out any inhibitory effect of glycerol. * statistically significant induction (*P* < 0.01, paired t test) in the lymphocyte proliferation when compared to negative control

Assessment of cytotoxicity of purified ILs was necessary to assess prior to their use in in vitro immunization. Cell density was increased significantly in case of both ILs when used with (IL-2 d-0.037 and IL-4 d-0.047; negative control d-0.029) or without glycerol (IL-2 d-0.048 and IL-4 d-0.04; negative control d-0.029) (Fig. 4). By adding glycerol in this experiment we tested its toxic effect on the PBMCs in in vitro immunization. Non-toxicity of ILs resuspended in 20% glycerol was confirmed (Fig. 4).

### sdAb phage clones isolated from the library constructed from in vitro immunized B cell

Peripheral blood monocytes were stimulated with H-OspA. Throughout in vitro immunization cell viability was evaluated everyday microscopically, wherein all cells were viable (data not shown). Similarly, there was no reduction in the cell count during and at the end of in vitro immunization (e.g. in cells incubated with antigen initial cell count was 1 × 10^6^ cells/mL; cell count at the end of immunization was 5.2 × 10^6^ cells/mL).

**Fig. 5:**
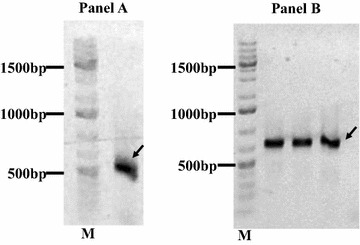
**a** *Arrow* indicates VHH fragment amplified from in vitro immunized lymphocytes. **b** *Arrow* indicates amplicons from the PCR performed to confirm insertion of VHH in pSex81 vector. Amplification was perfomed with pSex Insertom F and R primers. Note that this PCR add 150 nt to insert as primers are complimentary to vector (thus the molecular weights are higher ~700 bp)

**Fig. 6:**
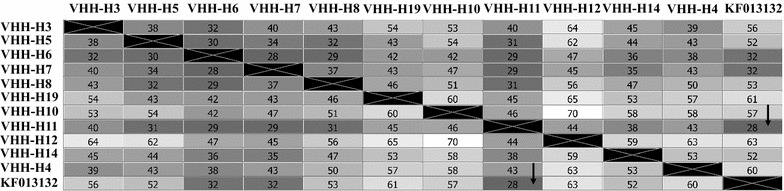
Sequence distance matrix built on in silico translated sequences of randomly picked clones from *E. coli* sdAb library (clones were designated as VHH-H3, 5, 7 etc.). KF013132—accession number of reference sequence from the Genbank. Number of amino acid residues not identical are presented in each square. Number of non-identical residues in matrix were >28 (*arrow*) indicating high divergence of the library

**Fig. 7:**
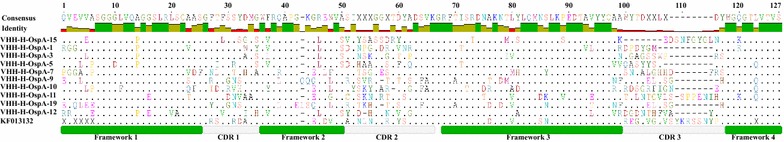
Amino acid sequence alignment of randomly picked clones from last titration of biopanning. VHH-H-OspA-1, 3, 5 etc.—representative amino acid sequence pertaining to each group (Clones with humongous sequences were clustered). *CDR* complementary consensus sequence. *Dots*—sequence homology. *Dashes*—gaps in the alignment. Amino acid letters appear in alignment in case of heterogeneity. Note the high level of heterogeneity in CDR3 followed by CDR2 and CDR1. Frameworks are relatively conserved

Total RNA isolated from antigen stimulated PBMCs was reverse transcribed and VHH fragments were amplified (Fig. 5). VHH fragments were ligated into the phagemid and a library for expression of sdAb on pIII was generated in *E. coli* XL-1 blue. A total of 1.3 × 10^6^ transfectants were obtained. Clones were picked randomly and sequenced with vector specific primers. Nucleotide sequences were translated in silico and amino acid sequences were used to plot distance matrix (Fig. 6). Number of non-identical residues (>28 residues) among the VHH sequences (Fig. 6) confirms the high diversity of the library. Hundred microliters of library was amplified in 2 × TY medium, phages were escaped with helper phage and the number of phages were calculated with phage titration, in which we obtained 2.04 × 10^16^ CFU/mL. Escaped phages (2 × 10^11^) were incubated with the same antigen used in in vitro immunization to capture the antigen binding phages (biopanning). Number of antigen binding phages in the eluate obtained from biopanning were calculated again with titration, wherein 1 × 10^4^ CFU/mL (approx. 1 × 10^4^ phages/mL) were counted. Randomly picked clones from the last titration were sequenced, translated in silico and amino acid sequences were aligned. Alignment was used to group clones with similar sequences. In total ten groups, each representing distinct full length VHH sequence were made (Fig. 7). Representative *E. coli* clone of each group was subjected for phage escaping, and phage clones were then used in phage dot blotting.

### Assessment of binding of sdAb to antigen

**Fig. 8:**
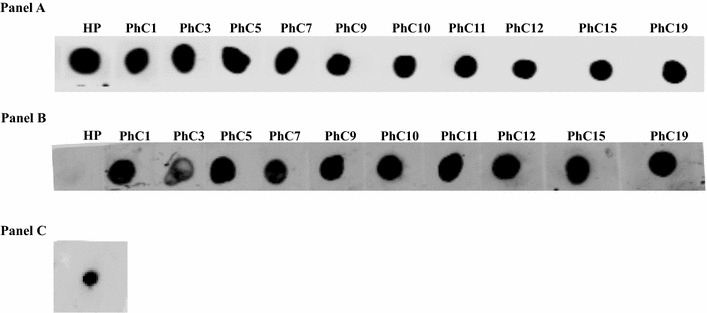
Qualitative phage dot blot. HP—helper phage spotted on the membranes. PhC1, 3, 5 etc.—escaped phages were spotted on the membranes. **a** Membrane with spotted phages was incubated with anti-pVIII antibody and then IRDye Detection antibody. **b** Membrane with phages was incubated with H-OspA-tagged with GFP and then anti-GFP antibody conjugated with C770 IRdye. **c** Input control for the antigen—H-OspA-tagged with GFP was spotted on the membrane and incubated with anti-GFP antibody conjugated with C770 IRdye. All secondary antibodies were conjugated with infra-red flurofores which enables quantification of signals in linear mode on infra red scanner (LI-Cor). Figures in the parenthesis indicates relative fluorescence unit (RFU)

Qualitative phage dot blotting confirmed the binding ability of sdAb expressed on phages to the antigen used in in vitro immunization (Fig. 8). To check whether the antigen interacts with phage coat proteins, helper phage was incubated with the antigen and subsequently with secondary antibody. No signal was noticed in this case (Fig. 8, panel B), which overrides the possibility of cross reactivity between antigen and sdAb carrying phage particle.

### Quantitative measurement of binding affinities

**Fig. 9:**
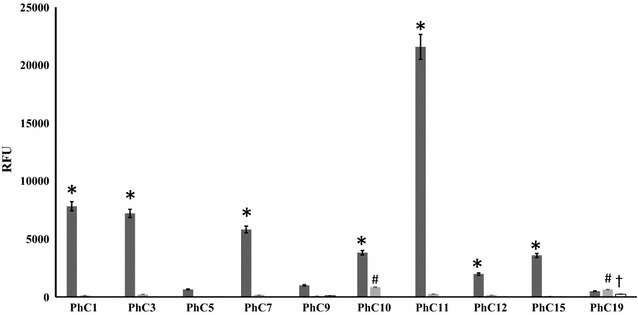
Quantitative phage ELISA. *Bar graph* depicts interaction of selected phage clones to H-OspA (*dark*-*grey*), non-related antigen SAA (*light*-*grey*), and to the wells without any antigen (*white bars*). PhC1-19—individual phage clones. Binding affinities of PhC5, PhC9, and PhC19 phage clones to H-OspA were significantly lower (*P* > 0.05) than other clones indicated with *asterisk*. Two phages, PhC10 and PhC19, showed weak non-specific binding to SAA antigen (#). PhC19 also showed weak affinity to plastware (†)

To assess strength of the binding between phages carrying sdAb and antigen, quantitative phage ELISA was performed (Fig. 9). The strongest affinity was found for clone PhC11 (RFU 21,586) followed by PhC1 (RFU 7830) and PhC3 (RFU 7210). Clones PhC5 (RFU 651; *P* = 0.5361, 95%). PhC9 (RFU 993; *P* = 0.4657, 95%) and PhC19 (RFU 496; *P* = 0.112, 95%) showed no significant binding affinity to H-OspA, whereas PhC10 and PhC19 showed weak non-specific binding to the non-related antigen SAA. None of the Phage clone, except PhC19 (RFU = 236), showed binding to plastic.

**Fig. 10:**
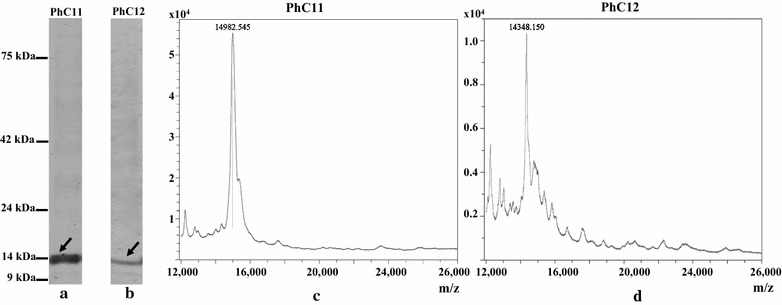
Soluble sdAbs. Presence and purity of soluble sdAbs were assessed by SDS-PAGE (**a**, **b** lanes). Molecular weights measured by MALDI–TOF (spectra **c**, **d**) of the purified sdAbs corresponded with theoretical molecular weights predicted in silico (PhC11—14.98 kDa, PhC12—14.34 kDa, predicted with Geneious Pro software). x-axis in MALDI spectra depicts m/z ratio while y-axis depicts intensity [a.u.]. Values 14,982.545 and 14,348.150 are in daltons

Two clones, PhC11 (RFU = 21,586) and PhC12 (RFU = 1976), which showed statistically significant strongest and weakest binding to H-OspA respectively were selected for measurement of K_D_ by bio-layer interferometry. Soluble sdAb produced from PhC11 and PhC12 (Fig. 10) showed K_D_ of 4.291 × 10E−6 and 7.905 × 10E−4, respectively (Table 2).

**Table 2:** Kinetic analysis of selected VHH clones

VHH	K_D_	Ka (1/Ms)	Kd (1/s)
PhC11	4.291E−6	1.366E4	5.861E−2
PhC12	7.905E−4	1.854E2	1.465E−1

## Discussion

With current interest in the applications of sdAbs in therapeutics and diagnostics, it is now necessary to overcome major hurdles in the isolation and synthesis of antigen-specific sdAb (e.g. time, cost, necessity of several camelid animals etc.). Thus, the primary aim of this study was set to establish a pipeline for cost effective and rapid production of sdAbs with antigen specific binding affinity. Here, as an antigen we used endothelial cell binding domain of OspA responsible for adhesion of *Borrelia* to brain microvascular endothelial cells, that further leads to translocation of pathogen into the brain [25].

The VHH, the core part of the sdAb, are unique in terms of size, epitope recognition, resistance to the heat and harsh pH, and show ability to penetrate into difficult areas or get through barriers (like blood brain barrier) [3, 17, 29, 30]. Existing pipelines for production of the sdAbs rely on the active immunization of animals, the first crucial step in any antibody synthesis. This approach is feasible in case of small animals (like rabbits, rats etc.), but cumbersome and costly in case of camelids. Another downside of active immunization is that it becomes extremely complicated when antigens are of high pathogenicity (infectious agents), toxicity or represents nonimmunogenic small molecules [31, 32]. Number of mRNA copies encoding antigen specific VHH are obviously higher in B cell repertoire of actively immunized animal than in naïve individual. This fact increases chances of getting more antigen specific nanobodies with stronger affinity. Several authors have attempted isolation of sdAbs from naïve libraries [33, 34], however struggled with lower antigen binding affinity or specificity. Moreover, they required extremely large sdAb libraries (e.g. 2.5–5 × 10^7^ and more). To overcome several disadvantages of active immunization or naïve libraries, we set to establish a pipeline for in vitro immunization followed by selection of antigen specific sdAb from sdAb-phage library.

Antigen-induced in vitro production of antibodies was attempted previously, however human PBMCs were used in their experiments and minimum of 8 days of lymphocyte culture was needed to obtain antibodies [22, 26]. Hitherto, there is no report that presents in vitro immunization of PBMCs from camelid animals. Two crucial components are required for successful in vitro immunization, the LLME (also called as Leu–Leu–OMe) and ILs. PBMCs contain lysosome-rich cytolytic T cell subpopulation, which is necessary to be removed from culture [35]. This subpopulation, sensitive to LLME, was thus treated before in vitro immunization in our study. Species-specificity of ILs, the second crucial component in in vitro immunization is important to address here. Species specificity of ILs was tested previously [36]. Authors found that human IL-2 efficiently stimulated proliferation of mouse T cells, however efficiency of mouse IL-2 to stimulated human T cells was 170-fold lower. It is noteworthy that, IL-4 (B cell stimulatory factor 1) is highly species specific. No B cell stimulatory activity was observed when mouse IL-4 was used to stimulate human lymphocytes and vice a versa [36]. Owing to this fact, we decided to produce IL-2 and IL-4 specific for the given camelid species. The ability to stimulate lymphocyte proliferation by recombinant ILs produced in our study was evident (Fig. 4).

Despite enormous advantages over other methods of antibody production, in vitro immunization may suffer from some inconveniences such as the IgM/IgG ratio, because this method tends to favor IgM isotypes. In the present study B cells were exposed to H-OspA only long enough to recognize the antigen and induce synthesis of mRNA copies that encode antigen-specific sdAbs. Shortening of the lymphocyte culturing to 4 day (as used in the present work) does not give sufficient time to differentiate B lymphocytes into plasma cells, which are primarily associated with production of IgM isotypes antibodies [22].

Antibody phage display based on phagemid vector has been successfully developed and used in the recent years. Several modifications in phagemid backbone were made to simplify cloning and library construction [37–42]. pSex81 phagemid used in our study was specifically developed for cloning of immunoglobulin heavy and light chain gene fragments isolated from human or mouse [43]. It was then modified for expression of functional single-chain Fv antibody—pIII fusion proteins on the surface of M13 bacteriophages [44]. pSex81 was also used successfully for construction of naïve sdAb library [45] production of complete human IgG [46], and for directed protein evolution [47]. Transformation efficiency of pSex81 is declared ~1 × 10^8^ CFU/μg DNA with chemically competent *E. coli* (XL1-Blue). The efficiency we obtained was ~1 × 10^9^ CFU/μg DNA. High cloning and transformation efficiency is crucial aspect in sdAb library construction as it directly affects the size and diversity of library, and moreover, increases the chances of getting antigen specific antibody with high affinity. Size of the library obtained in the present study was 1.3 × 10^6^, which is within the required range suitable for capturing antigen specific sdAb from immunized library. The use of *Nco*I restriction site incorporated in pSex81 may obstruct subcloning. Random presence of *Nco*I in the CDRs results in production of fragmented VHH and thus increases redundancy of clones. Few of the transfectants sequenced in our study showed such fragmentation (e.g. absence of framework-1 or framework-4, results not presented). Choice of alternative rare restriction site like *Sfi*I can easily overcome this drawback. As far as the diversity of library is concerned, sequence distance matrix plotted on VHH sequences obtained from our library and reference camelid VHH gene (KF013132) indicated complete non-specificity (i.e. high divergence, Fig. 6) of the library.

The affinities of soluble sdAbs produced from PhC11 and PhC12 were in micromolar range. To increase the affinity, soluble antibodies can be produced in periplasmic space of *E. coli* [48, 49] to allow formation of disulfide bonds and higher stability, or in eukaryotic secretory expression system like *Leishmania tarentolae* to maintain the post-translational modifications. Another option of improving the affinity may be the mutational hotspot randomization [50] or forming dimers by making fusion with alkaline phosphatase [51].

## Conclusions

In summary, joining the in vitro immunization of camelid lymphocytes and the phage display offers many advantages. In vitro immunization enables the efficient expansion of antigen-specific B cells among the PBMCs from only 10 ml of blood that substantially reduces the cost required to maintain a camelid herd. It also avoids time-consuming and laborious work involved in the construction of an extremely diverse synthetic or naïve libraries to obtain antigen-specific sdAb. Moreover, various immunogens including infectious agents and cell surface antigens can be employed easily in in vitro conditions to sensitize PBMCs. The pipeline presented here can be used to enrich antigen-specific B cells in camelid PBMCs and obtain antigen-specific sdAbs in short period.

## Abbreviations

BBB: blood–brain barrier
HcAbs: heavy (H)-chain antibodies
HUVEC: human umbilical vein endothelium cells
OspA: outer surface protein A
PBMC: peripheral blood mononuclear cells
VH: variable region in heavy chain
VHH: variable region in heavy chain in camelids
